# Multimodality radionuclide imaging in fever of unknown origin presenting with a solitary spleen lesion

**DOI:** 10.1186/s43055-022-00788-y

**Published:** 2022-05-13

**Authors:** Luca Filippi, Oreste Bagni, Orazio Schillaci

**Affiliations:** 1 Department of Nuclear Medicine, Santa Maria Goretti Hospital, Via Canova 3, 04100 Latina, Italy; 2 Department of Biomedicine and Prevention, University Tor Vergata, Rome, Italy; 3 IRCCS Neuromed, Pozzilli, Italy

**Keywords:** Fever of unknown origin, Splenic lesion, ^18^F-FDG, PET/CT, White blood cell scintigraphy

## Abstract

**Background:**

Fever of unknown origin (FUO) still represents a serious challenge for clinicians, since it can be related to a wide spectrum of disorders, ranging from infections to malignancies. In this scenario, nuclear medicine can be of value to achieve a correct diagnosis both through positron emission computed tomography (PET/CT) and ^99m^Tc labeled hexamethylpropylene amine oxime (HMPAO) white blood cell (WBC) scintigraphy.

**Case presentation:**

We are presenting the case of 65-year-old male, who was referred to our hospital due to prolonged unexplained fever. He was submitted to abdomen ultrasonography (US) that did not disclose relevant pathological findings. Subsequently, he underwent PET/CT scan with ^18^F-fluorodeoxyglucose (^18^F-FDG) that revealed an area of increased tracer uptake in splenic inferior pole. In order to solve differential diagnosis between tumor and infection, he was submitted to ^99m^Tc-HMPAO WBC scintigraphy that resulted negative for sites of pathologic radiolabeled cells’ accumulation but revealed a photopenic area in the splenic inferior pole. The pattern of mismatched uptake between ^18^F-FDG PET/CT and ^99m^Tc-HMPAO WBC scintigraphy was considered highly suspicious for spleen tumor localization. The patient was scheduled for splenectomy and histology resulted positive for non-Hodgkin lymphoma (NHL) of diffuse large B cell type. After splenectomy, a further ^18^F-FDG PET/CT revealed the appearance of hypermetabolic hepatic lesions. The patient underwent chemotherapy with complete remission.

**Conclusion:**

Nuclear medicine provides valuable tools for differential diagnosis in FUO. In case of patients presenting solitary lesion of the spleen, the combined use of ^18^F-FDG PET/CT and ^99m^Tc-HMPAO WBC scintigraphy can provide relevant information to aid clinicians to a correct diagnosis.

## Background

Fever of unknown origin (FUO), defined as a fever of 38.3 °C (101°F) or higher for 3 or more weeks in otherwise healthy patients, with no identified causes after clinical and laboratory investigations, still represents a challenge for clinicians [1]. Tumors, infections and non-infectious processes are the most common causes of FUO. Nuclear medicine has been successfully applied in the field of FUO, both through positron emission computed tomography (PET/CT) with ^18^F-fluorodeoxyglucose (^18^F-FDG) and ^99m^Tc labeled hexamethylpropylene amine oxime (^99m^Tc-HMPAO) white blood cell (WBC) scintigraphy. In a meta-analysis comparing the various techniques of nuclear medicine applied for FUO diagnosis, ^18^F-FDG PET/CT yielded the best performance, being characterized by a sensitivity of 0.86 and a specificity of 0.52 [2]. However, the combined value of the 2 aforementioned imaging approaches has been rarely investigated.

## Case presentation

**Fig. 1:**
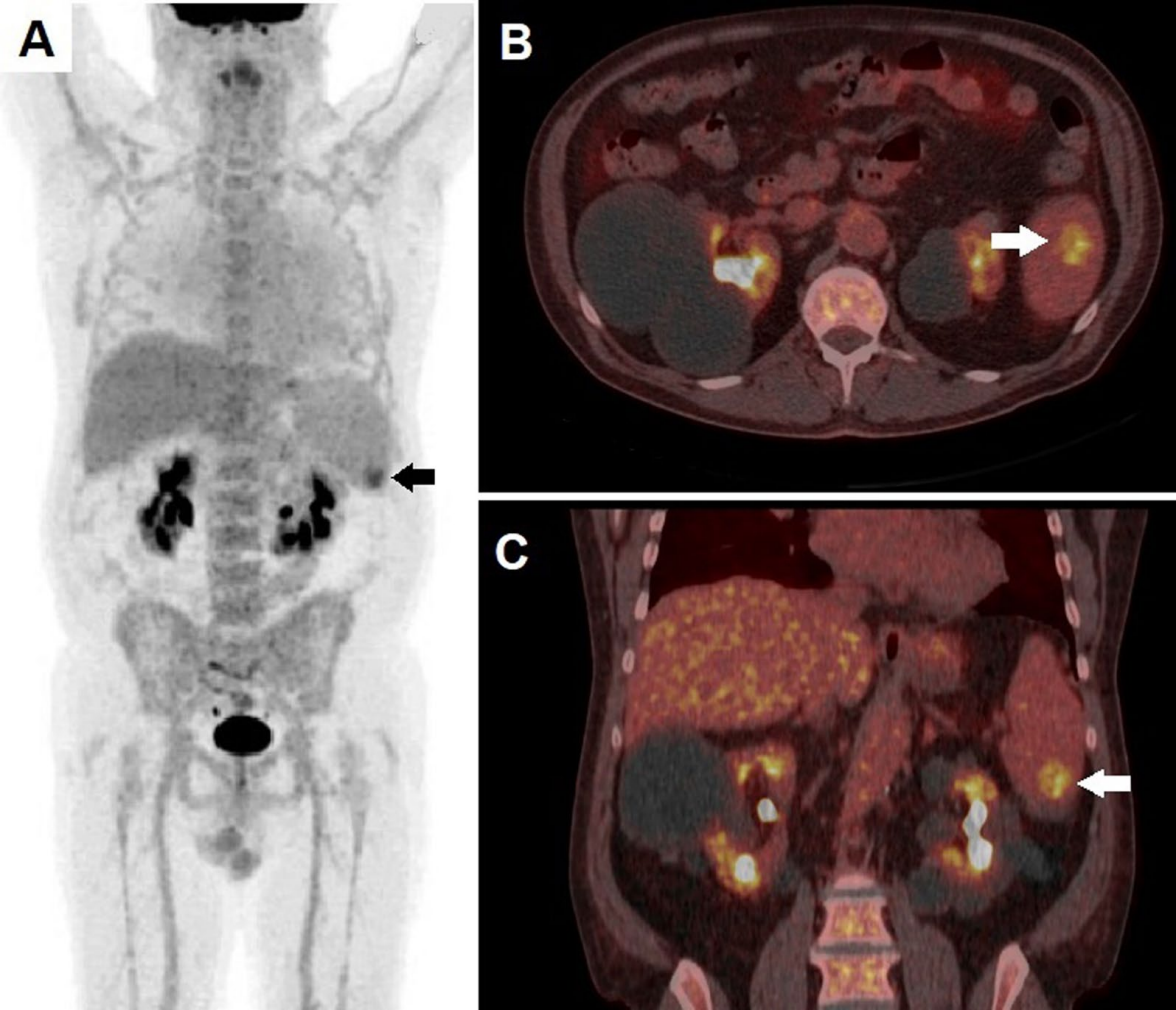
^18^F-FDG PET/CT Whole Body (**a**), performed at patient's admission, demonstrated an area of increased tracer incorporation (SUVmax 6.1) in the spleen inferior pole (black arrow), associated with diffuse bone marrow uptake. Fused axial (**b**) and coronal (**c**) PET/CT slices well depicted focal ^18^F-FDG accumulation (white arrow) within a hypodense area in the inferior splenic pole, with no evidence of meaningful spleen enlargement (splenic length 11 cm)

A 65-year-old male was admitted to our hospital in October 2020 with a history of 4-week fever (temperature of 38.5 °C) without specific symptoms. Clinical assessment and routine blood tests showed no significant abnormalities, chest radiograph and abdomen ultrasonography (US) resulted unremarkable too. He was submitted to antibiotic therapy with tigecycline with initial response and fever resolution. Two weeks later, the patient again experienced fever and was therefore submitted to ^18^F-FDG PET/CT. After i.v. administration of 3.7 MBq/kg of ^18^F-FDG, PET/CT was acquired from proximal thigh to skull base on a Siemens Biograph Vision 450 with an axial FOV of 197 mm using continuous-bed motion (FlowMotion®) with a bed speed of 1.5 mm/s (equivalent to approximately 90 s/bed position). Reconstruction was conducted with a TrueX + TOF algorithm and Gauss-filtered to a transaxial resolution of 2 mm at FWHM (full width at half maximum). Attenuation correction was performed using the low dose non-enhanced computed tomography data. PET/CT detected focally increased tracer incorporation within a hypodense area located in splenic inferior pole, with a maximum standardized uptake (SUVmax) value of 6.1 (Fig. 1).

**Fig. 2:**
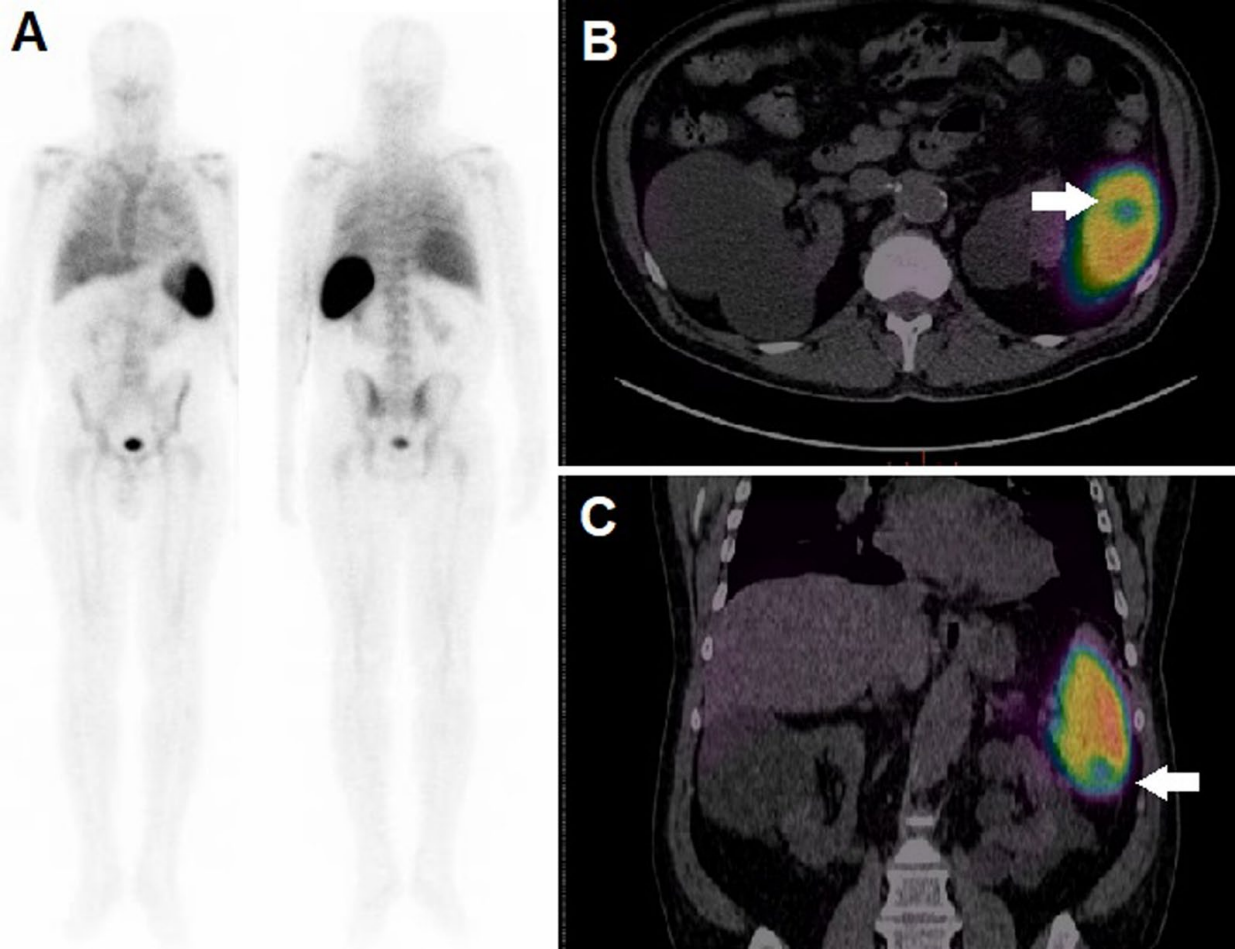
^99m^Tc-HMPAO WBC scan (**a**) in anterior (left) and posterior (right) view at 2 h p.i. did not detect any areas of pathological accumulation of the labeled cells. Fused axial (**b**) and coronal (**c**) slices demonstrated a photopenic area (white arrow) in the spleen inferior pole, corresponding to the hypermetabolic finding detected at ^18^F-FDG PET/CT

**Fig. 3:**
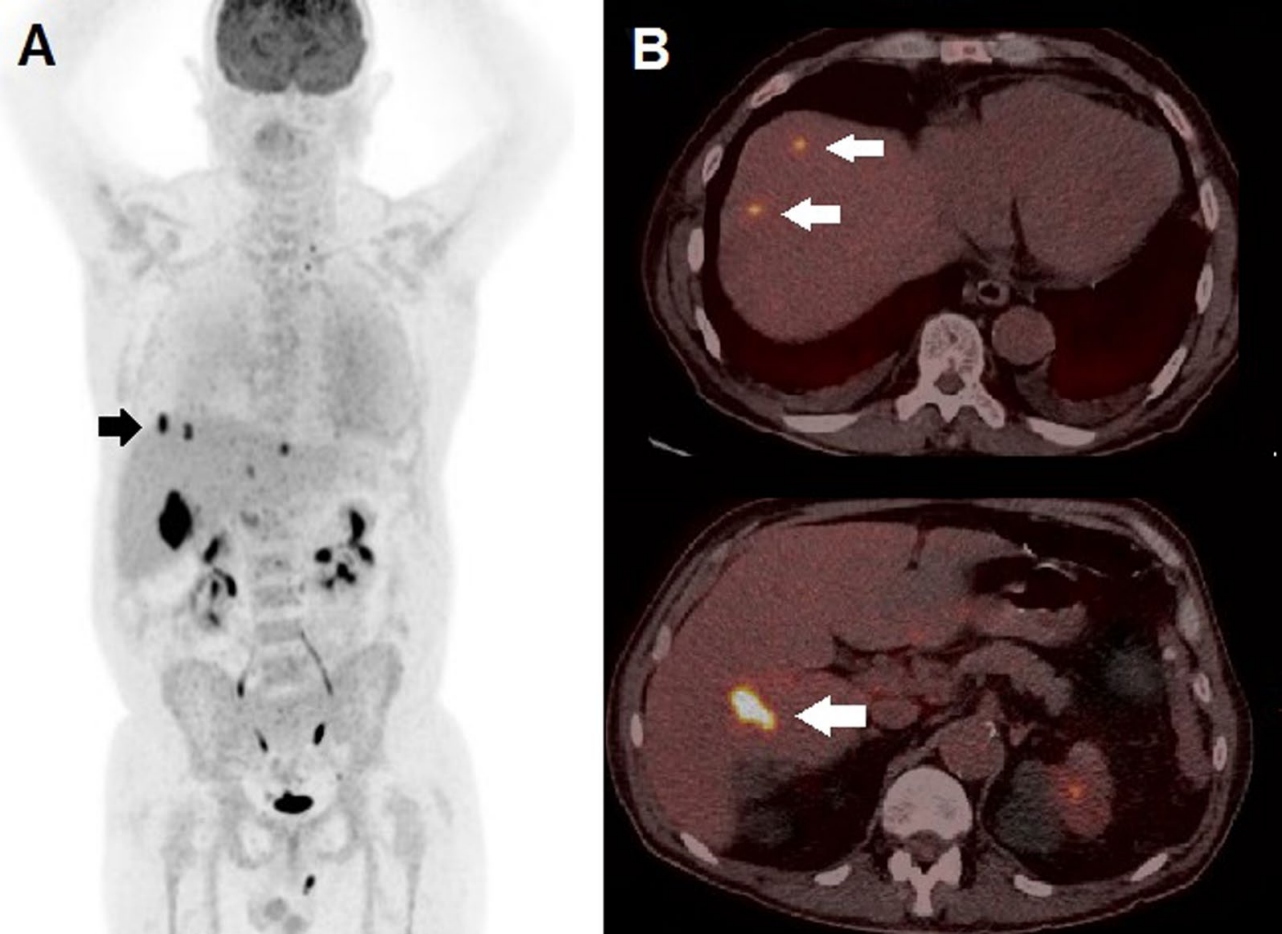
^18^F-FDG PET/CT Whole Body (**a**) performed after splenectomy demonstrated focuses of tracer uptake in the liver, as then detailed by fused PET/CT axial slices (**b**) that demonstrated hypermetabolic lesions (white arrows) in the hepatic dome (upper row) and in the 6th hepatic segment (lower row), with SUVmax of 25.3

In light of PET/CT's results, the patient was scheduled for oncology and infectivology consulting for further investigation on an outpatient basis. One week before performing consulting, as a result of COVID-19 pandemic outbreak, he was diagnosed with SARS-CoV-2, with consequent mild respiratory symptoms. He recovered from SARS-CoV-2 after 4 weeks without specific treatments; nevertheless, persistence of fever and meaningful weight loss (10 kg) were registered. Blood culture and bone marrow examination resulted negative. A total body CT scan did not show any relevant abnormality, except for the splenic hypodense area. In order to further exclude an infective nature of the aforementioned splenic finding, a ^99m^Tc-HMPAO WBC scintigraphy was carried out. After blood sampling, leukocytes were isolated and labeled as previously described [3]. The average labeling yield was 80% and the labeled cells were reinjected into the patient (administrated activity was 480 MBq). A dual-head, variable angle gamma-camera (Millennium VG, GE Healthcare) equipped with high-resolution low-energy collimators was used to obtain a whole body scan (anterior and posterior views) and multiple planar images of the abdominal region at 30 min and 2 h post-injection (p.i.) [3]. A SPECT study of the abdomen was also carried out at 2 h p.i., obtaining multiple views over 360° at a 30-s acquisition time per projection with an angular step of 3°. Reconstructed SPECT axial images were then co-registered and fused with the corresponding CT axials, acquired during PET/CT scan, through a dedicated software (Advantage 4.7, GE). ^99m^Tc-HMPAO WBC scintigraphy detected a photopenic area in correspondence of the splenic inferior pole and no other pathological findings (Fig. 2). The mismatched pattern of uptake among ^99m^Tc-HMPAO WB and ^18^F-FDG PET/CT scans was considered consistent with the suspicion of tumor location. The patient underwent laparoscopic splenectomy and histology resulted positive for non-Hodgkin lymphoma (NHL) of diffuse large B cell type. He was referred to the hematologists, who requested, before therapy start, a further ^18^F-FDG PET/CT that revealed the appearance of hepatic hypermetabolic lesions (Fig. 3). Afterward, the patient started chemotherapy according to R-CHOP regimen with complete response at follow-up PET/CT scan.

## Discussion

In spite of many advances in diagnosis and therapy, FUO still represents a clinical dilemma. In a recently published population-based cohort study [4], Søgaard et al. evaluated the risk of cancer in 6620 patients referred to hospital due to FUO between 1998 and 2017: 343 of them (i.e., about 5%) were diagnosed with cancer. Of note, the majority of cases were found to be hematological malignancies (HL, NHL, myelodisplastic syndromes, etc…). Primary splenic lymphoma has been rarely reported as a cause of FUO . Although spleen is commonly involved both at NHL and HL presentation [5], primary splenic lymphoma is a rare entity and is classified in four subtypes: splenomegaly without masses, military masses, scattered huge masses and solitary tumor [6]. Chen et al. reviewed 53 cases of splenic lymphoid tumors and found that all subjects presented a variable grade of splenomegaly [7].

^18^F-FDG PET/CT scan has a well-established role for diagnosis, staging and monitoring response to therapy in HL and NHL; however, primary lymphoma of the spleen has been sporadically described [8]. Takata et al. reported the case of 66-year-old woman, who was incidentally diagnosed with a hypodense splenic lesion at CT performed during the routinary work-up for hepatitis [9]. Since lesion's diameter increased at a further CT control after 4 months, ^18^F-FDG PET/CT was performed, disclosing a focal area of highly increased tracer uptake (SUVmax 12.7) in the splenic lesion, that resulted primary splenic lymphoma at histological examination after surgery. More recently, Sun and colleagues described a case of FUO due to primary splenic lymphoma in a 59-year-old male who was submitted to several clinical and instrumental investigations, among whom ^18^F-FDG PET/CT scan that revealed an enlarged spleen, characterized by diffusely increased tracer incorporation [10].

In our case, the pattern of ^18^F-FDG distribution was very similar to that described by Takata and colleagues [9], although with a less intense tracer incorporation within splenic lesion. The absence of B symptoms except for fever and weight loss, aside the negative findings of bone marrow examination, deserved for further investigations before scheduling patient for splenectomy. To the best of our knowledge, this is the first report describing the mismatched pattern of uptake of ^18^F-FDG PET/CT and ^99m^Tc-HMPAO WBC scans in a primary spleen lymphoma. ^99m^Tc-HMPAO WBC scintigraphy is widely utilized for FUO diagnosis in clinical practice, for its high sensitivity and specificity for detecting infectious and inflammatory processes in bones and soft tissues [11, 12]. Furthermore, the utilization of hybrid SPECT/CT devices has further moved the field forward, allowing the comparison between scintigraphy and other imaging modalities [13, 14]. It has to be highlighted that a splenic photopenic area at ^99m^Tc-HMAO WBC scan has been previously described by Erba and coworkers in a patient with multiple septic sites and has been attributed to septic embolism to splenic parenchyma [15]. In our patient, according to the overall clinical and laboratory conditions, it was considered unlikely that the splenic photopenic area might be attributed to a septic embolism, but rather to a malignant, infiltrative process determining focal loss of spleen functioning parenchyma.

## Conclusions

Nuclear medicine provides valuable tools for differential diagnosis in FUO. In case of patients presenting solitary lesion of the spleen, the combined use of ^18^F-FDG PET/CT and ^99m^Tc-HMPAO WBC scintigraphy can provide relevant information to aid clinicians to a correct diagnosis.

## Data Availability

Not applicable.

## Abbreviations

FUO: Fever of unknown origin
PET/CT: Positron emission computed tomography
HMPAO: Hexamethylpropylene amine oxime
WBC: White blood cell
